# Supplementary figures and images for: Altitude, not potential larval habitat availability, explains pronounced variation in Plasmodium falciparum infection prevalence in the western Kenya highlands
Source: PLOS Glob Public Health. 2023 Apr 17;3(4):e0001505. doi: 10.1371/journal.pgph.0001505 (PMC10109483; doi:10.1371/journal.pgph.0001505)

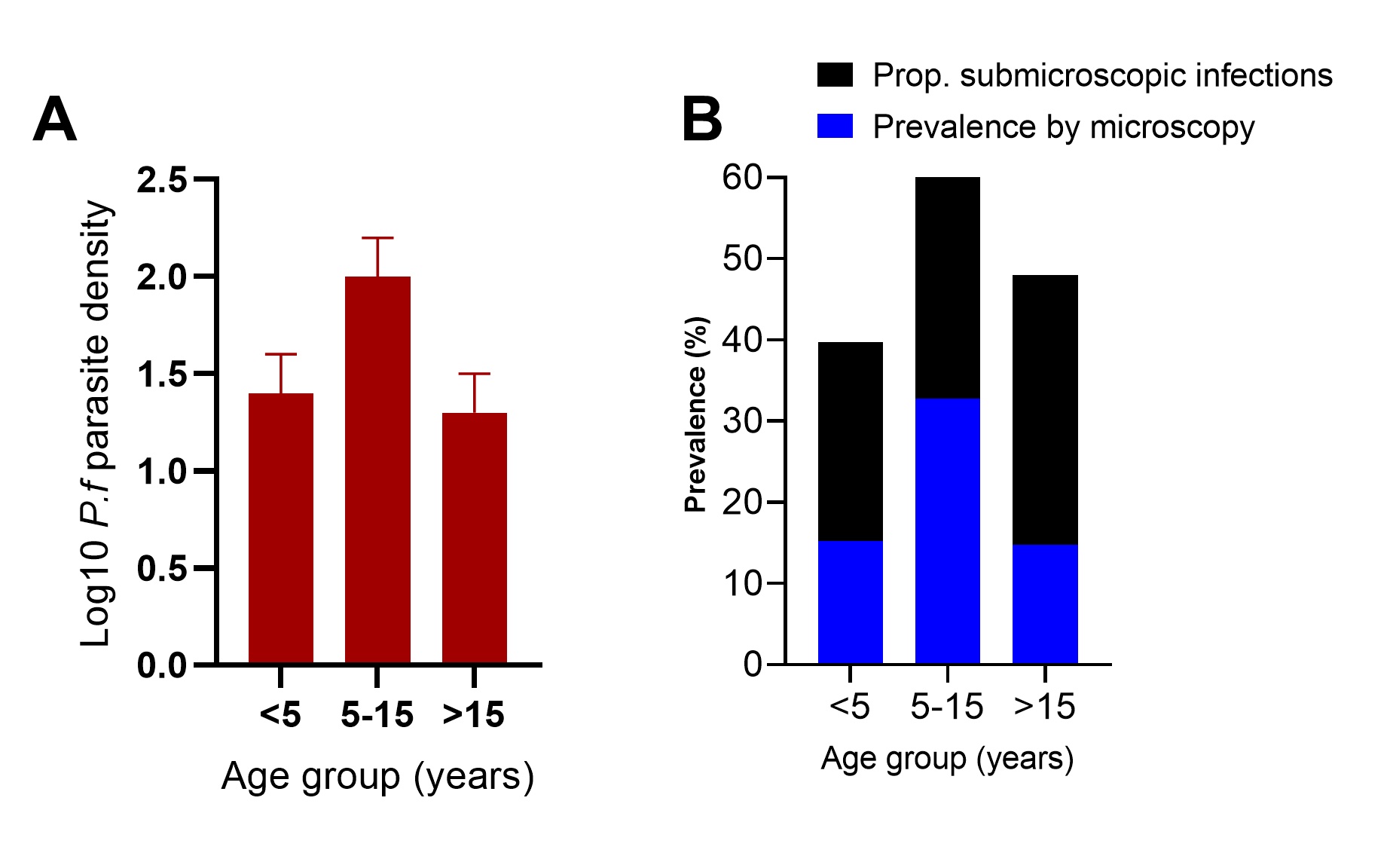

Supplement: S1 Fig — Age trends in P. falciparum density (A) and prevalence (B) by qPCR, and proportion of submicroscopic infections. Error bars in panel A shows standard errors of the geometric mean. In panel B, the proportion of submicroscopic infections is shown in addition to prevalence. (TIF) [file pgph.0001505.s001.tif]

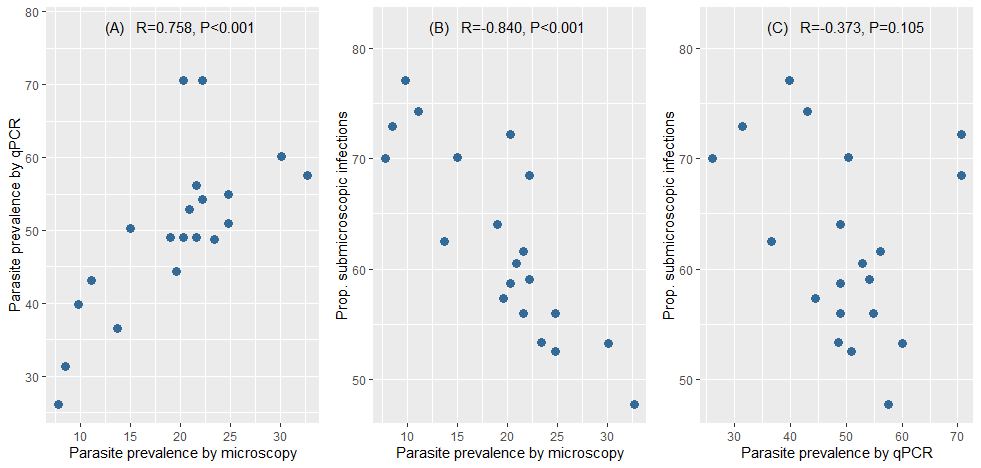

Supplement: S2 Fig — Each dot represents a cluster. R values are calculated by spearman correlation. (TIF) [file pgph.0001505.s002.tif]

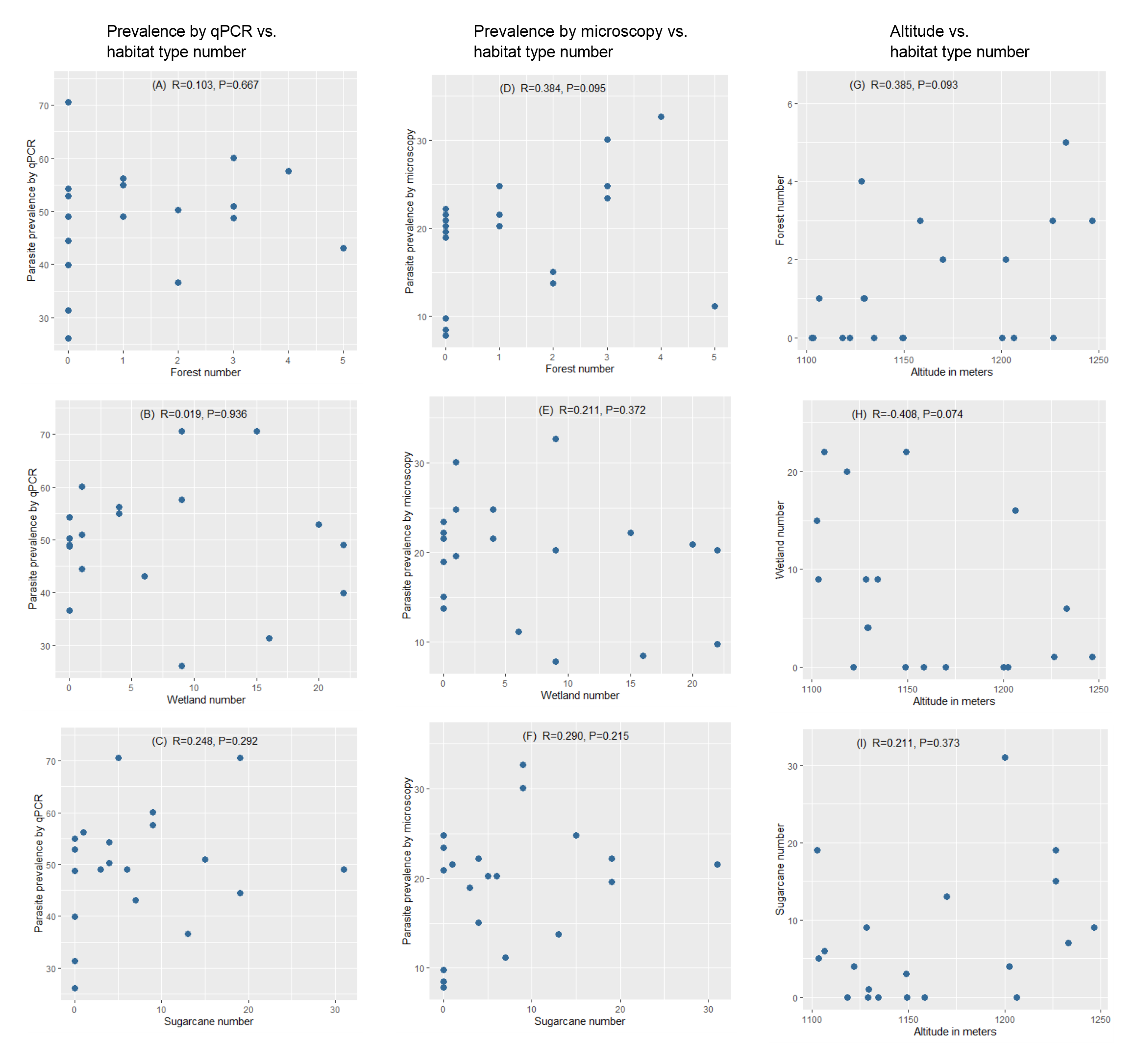

Supplement: S3 Fig — (TIF) [file pgph.0001505.s003.tif]
