# Supplementary material for: Altitude, not potential larval habitat availability, explains pronounced variation in Plasmodium falciparum infection prevalence in the western Kenya highlands
Source: PLOS Glob Public Health. 2023 Apr 17;3(4):e0001505. doi: 10.1371/journal.pgph.0001505 (PMC10109483; doi:10.1371/journal.pgph.0001505)
Supplement: Attachment — Submitted filename: Oduma_Response to reviewers .docx [file pgph.0001505.s005.docx]

We thank the reviewers for their detailed comments and recommendations for improvement. Their comments are in black, our answers in blue.

Journal Requirements:

1. Please review your reference list to ensure that it is complete and correct. If you have cited papers that have been retracted, please include the rationale for doing so in the manuscript text or remove these references and replace them with relevant current references. Any changes to the reference list should be mentioned in the rebuttal letter that accompanies your revised manuscript. If you need to cite a retracted article, indicate the article’s retracted status in the References list and also include a citation and full reference for the retraction notice.

2. Please amend your detailed Financial Disclosure statement. This is published with the article. It must therefore be completed in full sentences and contain the exact wording you wish to be published.

a. State what role the funders took in the study. If the funders had no role in your study, please state: “The funders had no role in study design, data collection and analysis, decision to publish, or preparation of the manuscript.”
b. If any authors received a salary from any of your funders, please state which authors and which funders.

If you did not receive any funding for this study, please simply state: “The authors received no specific funding for this work.”

3. Figure 2: Please confirm (a) that you are the photographer; or (b) provide written permission from the photographer to publish the photo(s) under our CC-BY 4.0 license.

The manuscript author is the photographer

4. Fig 1: please (a) provide a direct link to the base layer of the map (i.e., the country or region border shape) and ensure this is also included in the figure legend; and (b) provide a link to the terms of use / license information for the base layer image or shapefile. We cannot publish proprietary or copyrighted maps (e.g. Google Maps, MapQuest) and the terms of use for your map base layer must be compatible with our CC-BY 4.0 license.

Note: if you created the map in a software program like R or ArcGIS, please locate and indicate the source of the basemap shapefile onto which data has been plotted.

The map was created in ArcGIS.

Source of the shapefile: <https://open.africa/dataset?q=Kenya>

If your map was obtained from a copyrighted source please amend the figure so that the base map used is from an openly available source. Alternatively, please provide explicit written permission from the copyright holder granting you the right to publish the material under our CC-BY 4.0 license.

Reviewer #1: In this fine manuscript, Oduma and colleagues show that the number of potential breeding sites per village and next (<250 m) to households does not predict malaria prevalence measured during the dry season in the Western Kenya highlands. They show, however, that village altitude is a significant predictor of malaria prevalence in these same populations.

As the authors acknowledge, the main limitation of this study is that "potential" larval habitats have not been screened for the actual presence of anopheline larvae. In other works, the negative results may be associated, at least in part, with the unsurprising difficulty in accurately identifying larval habitats in the study site.

Another limitation arises from the cross-sectional nature of the study. Both exposure (e.g., proximity to larval habitats) and outcomes (presence of malaria parasites detected by microscopy or PCR) were measured at a single time point. The reader may wonder whether larval habitat availability during the dry season might predict, for example, malaria transmission in the beginning of the next rainy season. The reader may also wonder whether the proportion of submicroscopic and asymptomatic infections found during the dry season would be similar during the rainy season. Some further discussion of these limitations might be welcome.

We thank the reviewer for these comments and agree to the limitations. We have added the following paragraph to the discussion (lines 449-457):

“As a further limitation, habitat density and prevalence were determined at only one time point each. It is possible that habitat density predicts transmission intensity at the beginning of the rainy season, but that this effect wanes until the end of the main transmission season, when samples for the current study were collected. Of note, a previous study in western Kenya found moderate differences in prevalence between the dry and the wet season (17.5% vs. 13.5% by qPCR), i.e. less pronounced variation than among clusters in the present study [50]. Further research is needed to determine whether small-scale spatial variation in prevalence is maintained throughout seasons, and whether habitat density and prevalence correlate during some times of the year only.”

The authors have collected information regarding the presence of malaria related clinical symptoms 2 days prior to sample collection (lines 146-147), but I was not able to find any description of the proportion of infections that were symptomatic and how these changes with age and parasite density. I understand that the symptoms covariate was not included in adjusted models that aimed to identify correlates of infection, but at least a brief description of these findings might be useful.

We have added the following detail to lines 266-269:

“Among the 155 participants harboring malaria related clinical symptoms (i.e., symptomatic individuals), 22 (14.2%) were positive by microscopy and 64 (41.3%) by qPCR, while 69 (44.5%) did not carry any detectable parasites. The proportion of individuals reporting symptoms did not differ among age groups (*P*=0.907).”

There are a few additional minor points:

1. The authors say that "qPCR results were converted to varATS copies/μL using an external standard curve of ten-fold serial dilutions (5-steps) of 3D7 P. falciparum parasites quantified by droplet digital PCR (ddPCR)" (lines 199-201), but actually they analyze numbers of parasites (not of varATS copies) per microliter (e.g., lines 270-274). Did they simply assumed that each parasite genome has 20 varATS copies?

The reviewer is correct. We quantified parasites for the standard curve using a single-copy gene, and generated our curve using this quantification. Thus, there was no need for any additional calculations. We clarified this as follows (lines 203-204):

“Absolute parasite densities were obtained using an external standard curve of ten-fold serial dilutions (5-steps) of 3D7 *P. falciparum* parasites quantified by droplet digital PCR (ddPCR) [49].”

2. Why have the authors used the threshold of 250 m to define the presence of larval habitats next to households?

We considered a radius that we consider feasible for potential vector control interventions, such as larviciding, that is targeted or reactive (e.g. in response to clinical cases). We added the following detail (lines 161-163):

“The chosen radius represents a potentially feasible area for targeted or reactive vector control. While we are not aware of trials of focal larval source management, other reactive vector control interventions applied similar geographical scales [34–37].”

3. The following sentence is unclear: "The median odds OR for households was 2.94 based on the qPCR-detected infections
and 2.44 based on the microscopy-detected infections" (lines 380-381). Most likely, the authors would like to say: "The median odds ratio (OR) for households...". However, only in the next sentence the authors explain what they mean by "OR for households"" "The farther away a median OR is from 1, the higher the odds a person would be infected with malaria if there is a positive case within the same household or the same cluster." As this is a quite interesting result, a clearer description would be quite useful.

We have clarified this section as follows (lines 383-389):
“Clustering at the household and cluster level was assessed using the GLMM. The farther away a median odds ratio (OR) is from 1, the higher the odds a person would be infected with malaria if there is a positive case within the same household or the same cluster. The median OR for clustering of infections in households was 2.94 based on qPCR, and 2.44 based on microscopy. The median OR for clustering of infections in clusters was 1.60 based on qPCR, and 1.51 based on microscopy. The median OR results suggest that there is clustering at both household and cluster levels, and that clustering across households is stronger than across clusters.”

4. "S1 text" legend is: "S1 text. Age trends in P. falciparum density and prevalence by qPCR, and proportion of submicroscopic infections. Submicroscopic infections were determined as infections detected by qPCR but not by microscopy.". However, this is a supplementary figure, not a text. The legend does not describe what error bars show in panel A (standard deviation? standard errors of the geometric mean?). Panel B is unclear to me. Blue bar segments show prevalence (%) by age, but I do not understand what the black bar segments show. I believe that the authors would like to show the proportion of infections (within the blue bars) that are submicroscopic; if this is correct, they should provide a stacked chart where the total bar height indicates malaria prevalence in each age group (between 40 and 60%) and the highlighted bar segment (e.g., black) shows the prevalence of sub patent infections in each age group.

We agree that panel B was poorly designed. We redesigned it using stacked bars. We added the following clarifications to the legend. (lines 479-481):

“S1 Figure. Age trends in *P. falciparum* density (A) and prevalence (B) by qPCR, and proportion of submicroscopic infections. Error bars in panel A shows standard errors of the geometric mean. In panel B, the proportion of submicroscopic infections is shown.”

Reviewer #2: The MS is good as it has good information on the malaria cases that are unnoticed and sub clinical and submicroscopic this might give an idea of case burden. They may modify the title as the main finding seems to be different than the altitudes

Response: Compared to other variables, GLMM identified altitude as the main predictor for infection given its numerical nature i.e., every 10 m increase in altitude resulted in a 5.1% reduction in the odds of infection. Across the altitude range of over 200 meters, this is indeed the strongest predictor (resulting in a approximately 2.6-fold difference (1.051^19) across the altitude range studied. We thus believe the title is appropriate.

In Methods:
The study is only six month duration that too post rainy season then how the assessment of larval habitat were correlated?

Response: As detailed above in response to reviewer 1, we added a paragraph on this limitation to the discussion.

clusters were only based on altitudes?

Response: Clusters were designed to cover the study area evenly and to represent a range of altitudes. We added the following detail (lines 134-136):

“They were purposively selected based on their differences in altitude and designed to cover the study area evenly,”

As it suggests the study started during summer months it has to be added in the study duration (159-162)

Response: We added this detail (line 166-167):

“Therefore, we mapped habitats during the dry season in the months of February and March 2021”

In Results
the positives were among mostly asymptomatic?? (261-262 line)

Response: Yes, almost all positives were asymptomatic. This is expected in a region of medium-high transmission.

Table 1 shows that there is very less variation in the altitude therefore there may not be good correlation between cases and altitudes as it also depends upon internal house environment authors did not analyze as they have altitude in the title of their MS. However, they have correlated with the distances of breeding sites to households

Response: The title of our manuscript represents the analysis of the GLMM model. In this analysis, we included household characteristics and proximity to habitats. As explained above, altitude was the strongest predictor for the risk of infection in our model.

The Figures 3 and 4 are to be explained and could be reduced

Response: Figure 3 changed to supplementary figure (lines 485-487)

“S2 Figure. Relationships between prevalence by microscopy and qPCR, and proportion of submicroscopic infection across clusters. Each dot represents a cluster. R values are calculated by spearman correlation.”

In discussion
Asymptomatic cases are very high what is strategy for treating such cases who may not visit the facility (390 line)

Response: Currently, no strategies are in place in western Kenya to treat asymptomatic individuals. In general, strategies for targeting parasite reservoirs in the community include (a) screening of residents irrespective of clinical symptoms and administering treatment (i.e., mass screen-and-treat), (b) tracing, testing and anti-malarial treatment of household members of a positive malaria case identified from health facility, who test positive (i.e., reactive case detection), (c) monthly treatment of children under 5 years irrespective of clinical symptoms or infection during or shortly after the rainy season in areas that experience moderate-to-high but seasonal malaria transmission (i.e., seasonal malaria chemoprevention), or/and (d) administering antimalarial drug to individuals irrespective of clinical symptom or infection, especially in areas that are nearing elimination or are of low transmission (i.e., mass drug administration strategy). None of these are currently implemented in Kenya, and thus we feel it is not warranted to discuss them in our manuscript.

What were breeding sites of both species at what distance such sites are located? (411 lines)

Response: Preferred habitats are already described in lines 85-89:

*“Anopheles gambiae*, *An. arabiensis* largely share similar requirements for the larval environment, namely habitats with shallow waters exposed to sunlight [22,23]. *An. funestus* prefers a wide range of habitats including waterbodies that are mostly shady, permanent or semi-permanent, with floating or thick vegetation and algae [18,24,25].”

Local infections are possible in case indoor biting what are control efforts done at house levels by the government in such houses, and what are the process adopted by government on case report in the region any reactive surveillance occurs? what percent is the case reporting in the region? (419-420 lines).

Response: We added the following detail on current control interventions to the methods section, lines 129-131:

“In western Kenya region, bed nets are distributed every three years since 2004 by the National Malaria Control Program [29,30].”

Presence of male mosquitoes are also one of the indicators of nearby breeding habitat
As the different land forms supports different species of mosquitoes in vicinity please also provide any other species of mosquitoes encountered or found in breeding sites? (430-433 lines).

Response: No sampling of mosquitos was conducted as part of this study. Thus, we cannot comment on species composition, or presence of male mosquitos.
